# Charge‐Stabilized Nanodiscs as a New Class of Lipid Nanoparticles

**DOI:** 10.1002/adma.202408307

**Published:** 2024-11-14

**Authors:** Ivan S. Pires, Alexander Hostetler, Gil Covarrubias, Isabella S. Carlo, Jack R. Suggs, B.J. Kim, Andrew J. Pickering, Ezra Gordon, Darrell J. Irvine, Paula T. Hammond

**Affiliations:** ^1^ Koch Institute for Integrative Cancer Research Massachusetts Institute of Technology 500 Main Street Cambridge MA 02139 USA; ^2^ Department of Chemical Engineering Massachusetts Institute of Technology 21 Ames Street Cambridge MA 02139 USA; ^3^ Department of Biological Engineering Massachusetts Institute of Technology 25 Ames Street Cambridge MA 02139 USA; ^4^ Department of Materials Science and Engineering Massachusetts Institute of Technology Cambridge MA 02139 USA; ^5^ Ragon Institute of Massachusetts General Hospital Massachusetts Institute of Technology and Harvard University Cambridge MA 02139 USA; ^6^ Howard Hughes Medical Institute Chevy Chase MD 20815 USA

**Keywords:** anisotropy, layer‐by‐layer, lipid nanoparticles, nanodiscs, self‐assembly, tumor targeting

## Abstract

Nanoparticles have the potential to improve disease treatment and diagnosis due to their ability to incorporate drugs, alter pharmacokinetics, and enable tissue targeting. While considerable effort is placed on developing spherical lipid‐based nanocarriers, recent evidence suggests that high aspect ratio lipid nanocarriers can exhibit enhanced disease site targeting and altered cellular interactions. However, the assembly of lipid‐based nanoparticles into non‐spherical morphologies has typically required incorporating additional agents such as synthetic polymers, proteins, lipid‐polymer conjugates, or detergents. Here, charged lipid headgroups are used to generate stable discoidal lipid nanoparticles from mixed micelles, which are termed charge‐stabilized nanodiscs (CNDs). The ability to generate CNDs in buffers with physiological ionic strength is restricted to lipids with more than one anionic group, whereas monovalent lipids only generate small nanoliposomal assemblies. In mice, the smaller size and anisotropic shape of CNDs promote higher accumulation in subcutaneous tumors than spherical liposomes. Further, the surface chemistry of CNDs can be modified via layer‐by‐layer (LbL) assembly to improve their tumor‐targeting properties over state‐of‐the‐art LbL‐liposomes when tested using a metastatic model of ovarian cancer. The application of charge‐mediated anisotropy in lipid‐based assemblies can aid in the future design of biomaterials and cell‐membrane mimetic structures.

## Graphical abstract

Bottom‐up assembly of lipid‐based nanoparticles from mixed micelles containing di‐anionic lipids yield charge‐stabilized nanodiscs (CNDs). CNDs exhibit enhanced tumor accumulation compared to spherical liposomes. Surface chemistry modification via the layer‐by‐layer (LbL) technique improves tumor‐targeting properties compared to LbL‐liposomes. The insights presented can improve the assembly of delivery vehicles and expand the use of lipid‐based discoidal assemblies.

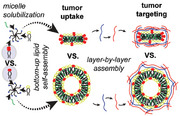

## 1 Introduction

Nanomedicine is a promising field that employs nanoscale materials for disease diagnosis and treatment.^[^ ^1^ ^]^ Nanoparticles (NP) can serve as carriers for insoluble drugs or as therapeutic delivery vehicles for substances that have inherently poor pharmacokinetic properties. Further, NPs can passively accumulate in certain tissues with high vascular permeability such as tumors, lymph nodes, sites of infection, or atherosclerosis.^[^ ^2^ ^]^ These effects may be enhanced through rational functionalization of NPs with targeting motifs. Accordingly, NPs serve to improve the effectiveness of a drug while also potentially mitigating side effects by accumulating in the disease site while sparing healthy tissue.

The size, shape, rigidity, and surface chemistry of NPs are all key parameters that regulate their pharmacokinetics, biodistribution, tissue penetration, and cellular uptake.^[^ ^3^, ^4^, ^5^, ^6^ ^]^ In the setting of cancer treatment, a major limitation of NPs is their poor tumor penetration as particles tend to accumulate perivascularly just outside tumor vessels with limited transport deeper into tumors.^[^ ^5^, ^7^ ^]^ To overcome this issue, recent studies have indicated that sub‐100 nm particles with high aspect ratio morphologies can significantly improve tumor penetration.^[^ ^5^, ^6^, ^7^, ^8^, ^9^, ^10^, ^11^ ^]^ Yet the most widely studied cancer nanomedicines have consisted of spherical nanoparticles.^[^ ^5^ ^]^ In the case of lipid‐based nanocarriers, this is in part due to the difficulties in controlling the self‐assembly of lipids into non‐spherical shapes. Some approaches to alter lipid‐based nanoparticles from spherical to discoidal have included the addition of lipid‐polymer conjugates, proteins, synthetic amphiphilic polymers, and mixtures of surfactant tail lengths.^[^ ^8^, ^12^, ^13^, ^14^, ^15^ ^]^ For example, we recently demonstrated that PEGylated lipid nanodics (LNDs) have significantly greater tumor penetration and accumulation in vivo compared to state‐of‐the‐art spherical liposomes.^[^ ^8^ ^]^ However, the use of polymer conjugates such as polyethylene glycol (PEG) or other additives to induce disc formation may introduce additional challenges such as increased immunogenicity, complement activation, or potential for allergic or hypersensitivity reactions.^[^ ^15^, ^16^, ^17^, ^18^ ^]^

In this study, we employed a bottom‐up approach to generate a new class of lipid nanodiscs starting from detergent micelles. While dilution of lipid/detergent mixture (mixed micelles) is a commonly used method to form liposomes,^[^ ^19^ ^]^ we discovered that the lipid head group charge could stabilize lipid nanodisc structures depending on the buffer ionic strength or the lipid headgroup charge valency. We show that using tangential flow filtration (TFF), a scalable size‐based separations technique, we can remove excess detergent to yield discoidal lipid nanoparticles which we termed charge‐stabilized nanodiscs (CNDs).^[^ ^20^ ^]^ We validate that CNDs have better tumor accumulation properties than liposomes. Further, we exploit the increased NP surface area of discoidal assemblies to modify CNDs with the layer‐by‐layer (LbL) technique to further improve their tumor targeting properties in a metastatic model of ovarian cancer.

## 2 Results and Discussion

### 2.1 Monovalent Lipids Fail to Generate Charged Nanodiscs Stable in Physiological Ionic Strength Solutions

**Figure 1:**
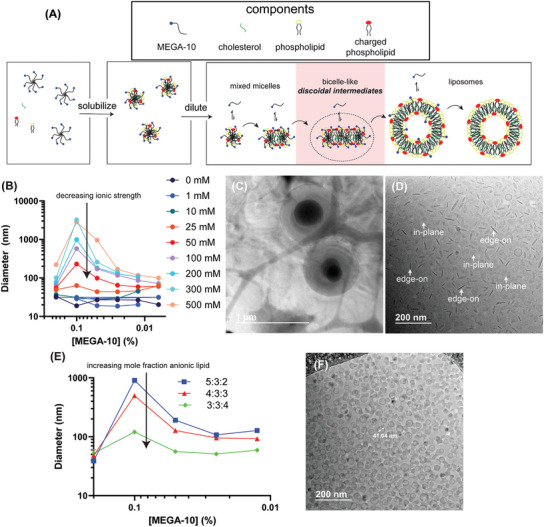
Charge‐stabilized nanodiscs (CND) with monovalent lipids are unstable in physiological ionic strength media. A) Schematic process for dilution of mixed micelles to generate discoidal lipid assemblies or liposomes. B) Lipid/detergent micelles (10 mg mL^−1^ of 6:3:1 molar mixture of DSPC:cholesterol:POPG in 10% MEGA‐10) in 10 mm HEPES buffer with different concentration of NaCl were diluted to indicated final MEGA‐10 concentrations and particle sizes (hydrodynamic Z‐avg) were assessed by DLS. C,D) CryoTEM micrographs of purified samples from dilution of lipid/detergent micelles using C) 200 mm NaCl and D) 0 mm NaCl. E) Intensity‐weighted diameter (Z‐avg) of PBS‐diluted lipid/detergent micelles containing 10 mg mL^−1^ of lipids in 10% MEGA‐10 composed of either 5:3:2, 4:3:3, or 3:3:4 molar ratios of DSPC:cholesterol:POPG. F) Representative cryoTEM micrograph of a 3:3:4 molar composition (DSPC:cholesterol:POPG) of mixed micelles allowed to equilibrate at 0.05% MEGA‐10 overnight then purified via TFF to remove MEGA‐10.

Upon dilution of mixed micelles (lipid/detergent mixtures) to below the detergent critical micelle concentration (CMC), liposomes are generally formed.^[^ ^19^, ^20^ ^]^ Given that liposome formation from mixed micelles occurs through discoidal intermediates^[^ ^21^, ^22^ ^]^ (**Figure** **1**A), we theorized that we could stabilize these intermediates by increasing electrostatic repulsion between lipid assemblies, and thereby prevent particle disc coalescence into liposomes. To evaluate this idea, we used buffers with decreasing ionic strength to dilute an anionic mixed micelle mixture [6:3:1 molar ratio of 1,2‐distearoyl‐sn‐glycero‐3‐phosphocholine (DSPC), cholesterol, and 1‐palmitoyl‐2‐oleoyl‐sn‐glycero‐3‐phospho‐(1′‐rac‐glycerol) (POPG) solubilized at 10 mg/mL in 10% (w/v) of the detergent N‐decanoyl‐N‐methylglucamine (MEGA‐10)] to various final MEGA‐10 concentrations and allowed them to equilibrate overnight (see Figure S1, Supporting Information, for chemical structure of each component). MEGA‐10 was chosen due to its high critical micelle concentration (CMC) which facilitates detergent removal, and its safe use for the production of NPs currently undergoing clinical evaluation.^[^ ^23^ ^]^

Dynamic light scattering (DLS) analysis of samples appeared to indicate that no liposomes were forming at low ionic strength conditions (<25 mm NaCl), due to the lack of particle size change upon dilution of lipid/MEGA‐10 solutions below the MEGA‐10 CMC (≈0.2–0.1 wt%,^[^ ^24^ ^]^ Figure 1B). By contrast, at higher ionic strengths, liposomes appeared to form which had increasing size as ionic strength increased, consistent with reduced electrostatic repulsion enabling increased particle coalescence. We next used cryogenic transmission electron micrography (cryoTEM) to evaluate sample morphology differences induced by changes in buffer ionic strength. Lipid/detergent mixtures were diluted to 0.1% MEGA‐10 in low (0 mM) or high (200 mM) NaCl for self‐assembly for 18 h, then further diluted to 0.02% MEGA‐10 before purification to fully remove detergent via tangential flow filtration (TFF). This incubation time was previously found to be required for full detergent partitioning into the aqueous phase for liposome assembly.^[^ ^20^ ^]^ High‐pressure liquid chromatography (HPLC) coupled with an evaporative light scattering detector (ELSD) confirmed the removal of >99.9% of detergent from samples via this approach (Figure S2A, Supporting Information). CryoTEM imaging confirmed that in high ionic strength buffers, unilamellar liposomes were formed (Figure 1C). However, particles diluted with low ionic strength buffer appeared to be primarily discoidal assemblies (captured either in the plane or viewed edge‐on, Figure 1D). This high homogeneity was surprising as previous work has only generated heterogeneous mixtures of liposomes, micelles, and discs with monovalent charged headgroups even when 80% of the lipids were charged in the composition.^[^ ^25^ ^]^ For biomedical applications, particles require colloidal stability; unfortunately, these species coalesced into liposomes when exposed to a physiological buffer – 10 mM pH 7.4 phosphate‐buffered saline (PBS) – leading us to term these as low ionic strength charge stabilized nanodiscs (lisCND, Figure S2B,C, Supporting Information).

To generate discoidal particles that would be stable in physiological buffers, we attempted to increase the mole fraction of anionic lipid instead of reducing the buffer ionic strength to potentially increase particle‐particle charge repulsion in high ionic strength solutions. We generated MEGA‐10 mixed micelles with molar lipid compositions of 5:3:2, 4:3:3, and 3:3:4 of DSPC:cholesterol:POPG and diluted them with PBS. The formulation with 3:3:4 yielded sub‐100 nm particles when diluted below the MEGA‐10 CMC, which would be indicative of reduced particle coalescence and potential discoidal assemblies (Figure 1E). We thus purified particles generated at 0.05% MEGA‐10 (Figure S2D, Supporting Information), but cryoTEM revealed that the sample was composed entirely of very small unilamellar liposomes (Figure 1F), indicating that monovalent surface charges alone were not sufficient to stabilize a disc morphology.

### 2.2 Increased Lipid Charge Valency Enables Synthesis of CNDs Stable in Physiological Buffers

We theorized that charged lipids promoted the formation of nanodiscs through lipid‐lipid repulsion at the disc edges, similar to how PEGylated lipids stabilize the edges of LNDs when a sufficient amount of PEGylated lipid is incorporated into the bilayer.^[^ ^26^ ^]^ In this scenario, increasing ionic strength would screen the stabilizing charges and destabilize the disc morphology. To overcome this issue, we posited that lipids containing two charges in close proximity could increase the lipid headgroup charge density and enable nanodiscs to remain stable in physiological ionic strength buffers.

**Figure 2:**
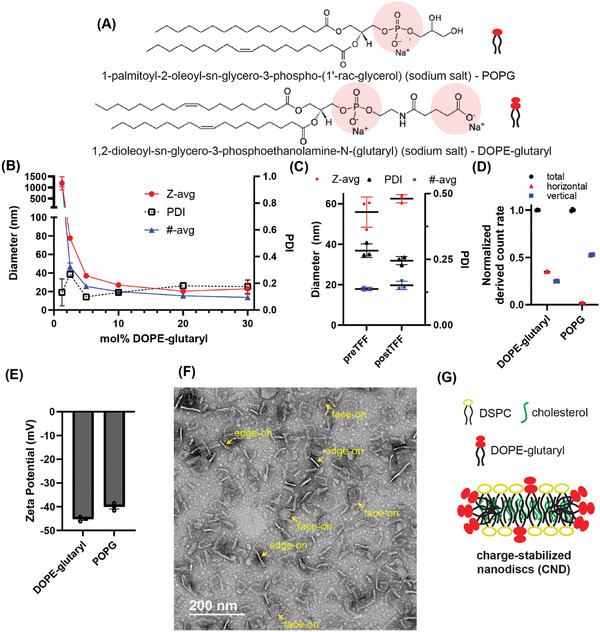
Charge density of anionic lipid DOPE‐glutaryl enables synthesis of CNDs stable at physiological ionic strength buffers. A) Chemical structure of POPG and DOPE‐glutaryl. B) DLS intensity weighted size (Z‐avg), number‐weighted average size (#‐avg), and polydispersity index (PDI) of particles assembled from varying compositions of DPSC, cholesterol, and DOPE‐glutaryl diluted in PBS to 0.1% MEGA‐10 concentrations (all samples contained 30 mol% cholesterol and the mol% indicated of DOPE‐glutaryl with the remainder being DSPC). C) DLS Z‐avg, #‐avg, and PDI of 10 mol% DOPE‐glutaryl CNDs before and after TFF purification. D) DLS count rate with or without polarized light filters. E) Zeta potential of CNDs composed with 10 mol% DOPE‐glutaryl compared to extrusion‐based liposomes with 10 mol% POPG (both samples contained 30% cholesterol and 60% DSPC). F) Representative negative stain TEM (NS‐TEM) micrograph of purified CNDs from (C). G) Proposed structure of CNDs composed of DOPE‐glutaryl, DSPC, and cholesterol.

To test this idea, we replaced POPG with the di‐anionic headgroup lipid 1,2‐dioleoyl‐sn‐glycero‐3‐phosphoethanolamine‐N‐(glutaryl) (DOPE‐glutaryl, **Figure** **2**A), and tested mixtures where the mole fraction of cholesterol was fixed and the amount of DPSC replaced by the anionic lipid was varied. Using the same detergent‐dilution method for controlling lipid self‐assembly, we used PBS to dilute samples to 0.1% MEGA‐10, as we found that samples that had large size (>100 nm) in these conditions were indicative of disc coalescence and liposome formation. While the inclusion of low amounts of DOPE‐glutaryl (<2.5 mol%) yielded large particles (>100 nm), increasing the DOPE‐glutaryl content above 5 mol% gave small particles (Z‐avg < 50 nm, Figure 2B). To better characterize these assemblies, we synthesized a larger batch size of 10 mol% DOPE‐glutaryl NPs. After overnight incubation at 0.1% MEGA‐10 to allow self‐assembly and full detergent partitioning to the solution, the sample was further diluted to 0.02% MEGA‐10 and purified via TFF to remove the detergent. We confirmed that TFF could achieve >99.9% removal of MEGA‐10 from the particles (Figure S3A, Supporting Information) without substantially affecting the particle size, yielding particles with an expected physical size (based on DLS number‐average, #‐avg) of ≈20 nm (Figure 2C).

If these charged lipids were assembled into nanodiscs, we would predict a higher degree of anisotropy in light scattering from the particles.^[^ ^27^ ^]^ Indeed, we found that evaluating the contribution of polarized (vertical) versus horizontal (depolarized) scattered light to sample counts in dynamic light scattering, the particles formed with DOPE‐glutaryl had relatively high depolarized scattering compared to control spherical liposomes formed with 10% POPG (Figure 2D). However, similar to 10% POPG liposomes, the purified particles exhibited negative zeta potential (Figure 2E). Negative stain TEM imaging confirmed that DOPE‐glutaryl particles had a nanodisc morphology based on the presence of the disc‐like structures in characteristic “edge‐on” and “face‐on” orientations (Figure 2F).^[^ ^28^ ^]^ Given that these particles maintained a stable small ≈20 nm #‐avg size in a physiological buffer (PBS) even after prolonged storage at 4 °C (Figure S3B,C, Supporting Information), we termed them simply charge‐stabilized nanodiscs (CNDs) and show their proposed structure in Figure 2G.

### 2.3 CNDs have Higher Tumor Accumulation In Vivo Compared to Liposomes

**Figure 3:**
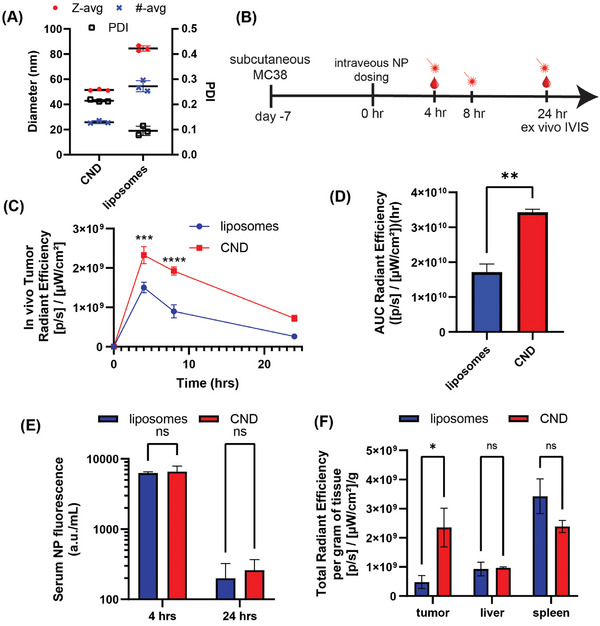
CNDs show greater tumor accumulation than liposomes in solid tumors. A) DLS Z‐avg, #‐avg, and PDI of purified CNDs composed with 10 mol% DOPE‐glutaryl compared to extrusion‐based liposomes with 10 mol% POPG (both samples contained 30% cholesterol and 60% DSPC). B) In vivo study timeline in which mice were inoculated subcutaneously with 10^6^ MC38 cells and then dosed intravenously on day 7 with 1 nmol of cyanine‐5 labeled NPs (1 mol%). C) Tumor radiant efficiency measured in vivo via IVIS. D) Area under the curve (AUC) or data from (H). E) Serum fluorescence of CNDs and liposome‐dosed animals at 4 and 16 h after dosing. F) Recovered radiant efficiency from tumor, liver, and spleen 24 h after dosing mice with either CNDs or liposomes. Error bars represent SEM (n = 3). Statistical comparisons in (C,E,F) were performed using two‐way analysis of variance (ANOVA), with Tukey's multiple‐comparisons test and an unpaired two‐tailed t‐test was performed for D. Asterisks denote p‐values: ^****^ *p* < 0.0001, ^***^ *p* < 0.001, ^**^ *p* < 0.01, ^*^ *p* < 0.05.

Anisotropic particles such as worm‐like micelles and nanodiscs have been reported to exhibit a higher degree of tumor accumulation than traditional spherical liposomes.^[^ ^5^, ^6^, ^7^, ^8^, ^9^, ^10^, ^11^ ^]^ To determine if CNDs could exhibit similar tumor uptake, we compared intravenous injection of fluorescently labeled anionic DOPE‐glutaryl CNDs to anionic POPG liposomes in mice bearing subcutaneous tumors (**Figure** **3**A,B). To validate that the fluorescently labeled lipids remained attached to the CNDs in vivo, we incubated the particles in 100% serum for 24 h at 37 °C and then used 100 kDa centrifugal filters to separate any free lipids from the CNDs. Less than 1% of free dye could be observed via this approach (Figure S4A, Supporting Information). While the liposomes were larger than CNDs, 50–60 nm liposomes are one of the most common NPs for drug delivery and we could not generate smaller liposomes without substantially altering their composition, consistent with the poor stability of liposomes <≈40 nm.^[^ ^29^ ^]^ As shown in Figure 3C,D, there was a significant increase in the tumor accumulation of CNDs compared to liposomes (Figure S4B, Supporting Information), even though levels of the two particle types in blood were similar over time (Figure 3E). Moreover, while there was no significant difference in NP accumulation in major clearance organs (liver and spleen), CNDs were able to preferentially accumulate in tumors compared to liposomes (Figure 3F; Figure S4C, Supporting Information). These results further validate that anisotropic structures such as CNDs can achieve better tumor accumulation in vivo compared to spherical state‐of‐the‐art liposomes.^[^ ^8^, ^9^, ^10^, ^11^, ^30^, ^31^, ^32^, ^33^, ^34^, ^35^, ^36^ ^]^

### 2.4 Deposition of Thin Polyelectrolyte Films via Layer‐by‐Layer (LbL) Technique Enables Increased Tumor Cell Association of LbL‐CNDs Compared to LbL‐Liposomes In Vitro

**Figure 4:**
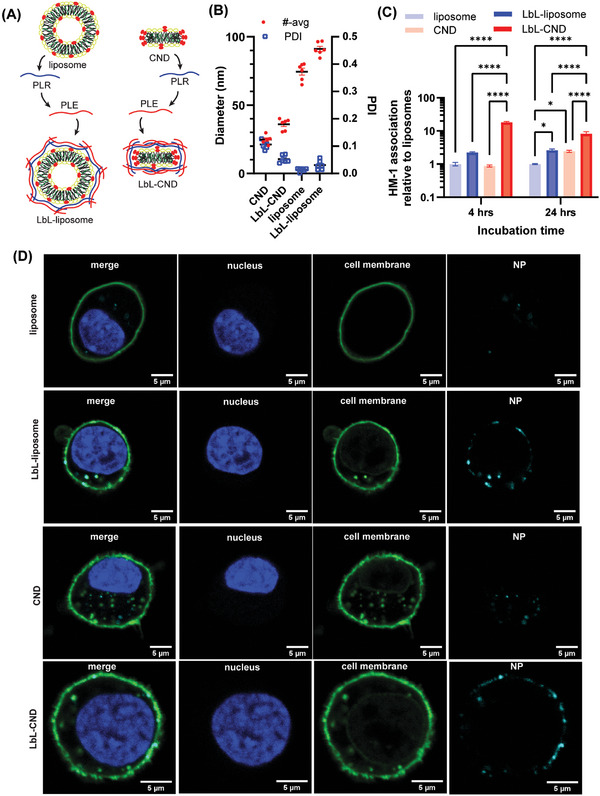
Deposition of polyelectrolyte layers composed of PLR and PLE onto CNDs enables improved association of CNDs with ovarian cancer cells in vitro. A) Schematic of LbL technique to generate LbL‐liposomes and LbL‐CNDs. B) Size and PDI of CND and liposomes before and after LbL modification. C) In vitro measurement of total HM‐1‐associated NP fluorescence relative to liposomes using a plate reader after 4 or 24 h of incubation. D) Confocal images of HM‐1 cells after 4 h of incubation with NPs. Error bars represent SEM. Statistical comparisons in C was performed using two‐way analysis of variance (ANOVA), with Tukey's multiple‐comparisons. Asterisks denote p‐values: ^****^ *p* < 0.0001, ^***^ *p* < 0.001, ^**^ *p* < 0.01, ^*^ *p* < 0.05.

Previous work has shown that the deposition of thin polymer films onto nanoparticles via the layer‐by‐layer (LbL) technique can enable controlled drug release and tumor targeting.^[^ ^37^, ^38^, ^39^ ^]^ The choice of polymer chemistry regulates the overall LbL‐NP properties, enabling a wide range of particle characteristics.^[^ ^40^ ^]^ Given that the association of LbL‐NPs with cells is a surface‐driven phenomena, we theorized that the increased surface area of CNDs could generate improved LbL‐NP formulations relative to spherical liposomes. We previously showed that depositing a bilayer of poly‐L‐arginine (PLR) and poly‐L‐glutamate (PLE) onto NPs promotes binding of the particles to the surface of ovarian cancer cells without triggering endocytosis, such that PLE‐coated NPs accumulate on the cell surface.^[^ ^40^, ^41^, ^42^ ^]^ Thus, we next attempted to layer CNDs with PLR and PLE and compared the resulting nanoparticles to layered liposomes (**Figure** **4**A). Prior to purification via TFF, excess polymer was used during each stage of LbL deposition to ensure rapid full surface coverage of CNDs and prevent particle aggregation.^[^ ^43^ ^]^

**Figure 5:**
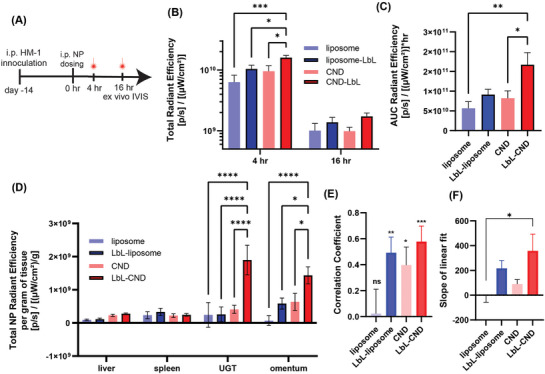
LbL‐CNDs NPs efficiently target metastatic ovarian cancer in vivo. A) In vivo timeline for treatment of fluorescently‐labeled NPs i.p. in ovarian cancer model. Mice were inoculated with 10^6^ HM‐1‐luc cells i.p. and dosed with NPs 14 days later. B) Total radiant efficiency of NP fluorescence from peritoneum. C) AUC of peritoneal fluorescence readings from (B). D) Ex vivo weight normalized NP fluorescence in liver, spleen, urogenital tract (UGT), and omentum. E) Spearman's correlation coefficient between weight‐normalized NP fluorescence and weight‐normalized BLI readings. Error bars (s.e.m.) derived from parameter estimates for each group. F) Slope of linear fit between weight‐normalized NP fluorescence and weight‐normalized BLI readings. Error bars (s.e.m.) represent variation between each animal in respective treatment groups (n = 4 mice/group). Statistical comparisons in B and D were performed using two‐way analysis of variance (ANOVA) one‐way ANOVA was used in C and F with Tukey's multiple‐comparisons test. Spearman's correlation significance for E was performed based on a t‐test analysis with the null hypothesis of no (r = 0) correlation. Asterisks denote p‐values: ^****^ *p* < 0.0001, ^***^ *p* < 0.001, ^**^ *p* < 0.01, ^*^ *p* < 0.05.

As expected, addition of the polyelectrolyte layers led to characteristic changes in zeta potential of both CNDs and liposomes as the polymers were layered (Figure S5A, Supporting Information). Both liposomes and CNDs exhibited increases in hydrodynamic size following layering, but maintained low PDIs (PDI < 0.2, Figure 4B). Consistent with prior findings for LbL‐coated nanoparticles, CND‐LbL particles were unstable in the presence of high ionic strength buffers,^[^ ^44^, ^45^, ^46^ ^]^ but did not show signs of major aggregation in the presence of serum (Figure S5B, Supporting Information). Following the incubation of fluorescently‐tagged particles with HM‐1 ovarian cancer cells, both LbL‐liposomes and LbL‐CNDs yielded the expected increase in cancer cell association relative to bare POPG liposomes (Figure 4C). However, LbL‐CNDs increased ovarian cancer cell binding by ≈3‐fold relative to LbL‐liposomes (Figure 5C). Interestingly, while lisCNDs (nanodisc structures formed from POPG formulations in low ionic strength) could also be modified via the LbL technique (Figure S5C, Supporting Information), only LbL‐CNDs presented an improved association with ovarian cancer cells in vitro relative to LbL‐liposomes (Figure S5D, Supporting Information). This could be due to the instability of lisCND which were found to have lower uptake than unlayered liposomes whereas CND had equal or higher uptake by ovarian cancer cells. Similar to the behavior we previously reported for anionic liposomes coated with PLR and PLE, confocal imaging revealed that “bare” CNDs were endocytosed by the tumor cells, while LbL‐CNDs remained at the cell surface for at least 4 h (Figure 4D).

### 2.5 Deposition of Thin Polyelectrolyte Films via Layer‐by‐Layer (LbL) Technique Enables Increased Tumor Cell Association of LbL‐CNDs Compared to LbL‐Liposomes In Vivo

Based on the improved in vitro ovarian cancer binding of LbL‐CNDs compared to LbL‐liposomes, we next examined the in vivo tumor‐targeting properties of LbL‐CNDs in a model of metastatic ovarian cancer. Before in vivo dosing of CNDs, we validated that these new NPs did not cause cytotoxicity in vitro on the model human embryonic kidney (HEK) 293 cell line and found no evidence of toxicity (Figure S6A, Supporting Information). Luciferase‐expressing HM‐1 cells were injected intraperitoneally (i.p.) into mice and allowed to establish for 14 days, followed by i.p. administration of fluorescent liposomes or CNDs, with or without the LbL coating (**Figure** **5**A).

Mice given LbL‐CNDs had significantly higher (≈2‐fold) in vivo peritoneal fluorescence 4 h after dosing compared to other groups indicating a better retention of NPs in the i.p. space (Figure 5B,C; Figure S6B, Supporting Information). While accumulation in the major clearance tissues (spleen and liver) was low, ex vivo measurement of total NP fluorescence in the main sites of metastasis development (i.e., omentum and urogenital tract, UGT^[^ ^47^ ^]^) revealed a significantly improved accumulation of LbL‐CNDs in tumor tissue with a 10‐fold or more increase relative to bare liposomes (Figure 5D; Figure S6C, Supporting Information). Further, as all peritoneal organs present some level of tumor burden which could be measured via bioluminescence intensity (BLI) readings, we analyzed the correlation between the tumor burden of an organ to its NP fluorescence reading. While bare liposome fluorescence did not correlate with tissue tumor burden, LbL‐liposomes showed a significant correlation, consistent with the tumor‐targeting properties of LbL‐coated NPs (Figure 5E). Moreover, both CND and LbL‐CND accumulation were correlated with tumor burden, albeit CND‐LbL treatment yielded a more confident fit. Indeed, LbL modification of CNDs increased the amount of NP accumulation per BLI reading ≈4‐fold (Figure 5F). These results indicate that combining the nanodisc morphology with an ovarian cancer‐targeting LbL coating substantially enhances the delivery of lipid nanoparticles to metastatic ovarian cancer.

## 3 Conclusion

Lipid nanoparticles are important delivery vehicles for current and new‐generation therapeutics. Here we have shown that through the rational disassembly of mixed micelles, we can stabilize nanodisc intermediates to generate a new class of lipid nanoparticles – charge‐stabilized nanodiscs (CNDs). We also found that assembly from detergent micelles enabled the synthesis of minute liposomes. While not the focus of the present paper, these minute liposomes with minimal polydispersity are close to the limit in size of nanoliposomal systems which have great potential for biomedical applications.^[^ ^48^ ^]^ Achieving such small liposome sizes (30‐40 m) via conventional methods has been challenging.^[^ ^49^ ^]^ Even with optimized conditions, previously reported limit‐size nanoliposomes have exhibited high polydispersity (PDI>0.1).^[^ ^48^, ^49^ ^]^

Importantly, CNDs demonstrated improved tumor accumulating properties compared to standard liposomes and could be surface modified via the LbL technique to further promote tumor targeting. Previous studies have shown that PEG, proteins, synthetic polymers, or mixtures of short and long‐tailed surfactants (such as detergents and lipids) can generate disc‐like structures.^[^ ^8^, ^12^, ^13^, ^14^ ^]^ However, these added components increase the risk of immunogenicity and allergen sensitivity and can alter the particle characteristics. In the case of detergent‐stabilized bicelles, they are limited by their sensitivity to dilution which did not occur with CNDs.^[^ ^13^ ^]^ While it has been shown that lipids with charged dendritic headgroups (>5*e*) can yield disc‐like micellar structures, they are limited to formulations in high‐resistivity water, require >50 mol% of the charged lipid in the composition, and need sonication to reduce particle size.^[^ ^25^, ^50^ ^]^ Through the readily scalable method presented here, the resulting CNDs were stable in a physiological buffer and required <10 mol% of a bivalent charged lipid. Even though CNDs lack the inner aqueous core of liposomes for the loading of therapeutics, drugs can be readily incorporated into lipid bilayer or via lipid‐drug conjugates.^[^ ^8^, ^51^, ^52^ ^]^

Taken together, we demonstrate that the novel CND assemblies have great potential for use in biomedical applications. CNDs may also be valuable in structural and functional studies of membrane proteins given the lack of synthetic constituents.^[^ ^53^, ^54^, ^55^ ^]^ Further, the techniques and discoveries presented here may facilitate the generation of new and more controlled lipid‐based assemblies.

## 4 Experimental Section

### Materials

1,2‐distearoyl‐sn‐glycero‐3‐phosphocholine (DSPC), Cholesterol, 1‐palmitoyl‐2‐oleoyl‐sn‐glycero‐3‐phospho‐(1′‐rac‐glycerol) (sodium salt) (POPG), 1,2‐dioleoyl‐sn‐glycero‐3‐phosphoethanolamine‐N‐(glutaryl) (sodium salt) (DOPE‐glutaryl), 1,2‐dioleoyl‐sn‐glycero‐3‐phosphoethanolamine‐N‐dibenzocyclooctyl (DOPE‐DBCO), 1,2‐distearoyl‐sn‐glycero‐3‐phospho‐(1′‐rac‐glycerol) (DSPG), and 1,2‐distearoyl‐sn‐glycero‐3‐phosphoethanolamine‐N‐(Cyanine 5) (DSPE‐cy5) were purchased from Avanti Polar Lipids. Poly‐L‐arginine (PLR) with a molecular weight (MW) of 9.6 kDa and poly‐L‐glutamic acid (PLE) with a MW of 15 kDa were purchased from Alamanda Polymers. N‐decanoyl‐N‐methylglucamine (MEGA‐10) and n‐octyl‐β‐d‐glucoside (octylglucoside) were purchased from Sigma Aldrich. For fluorescence measurements with lisCND samples, borondipyrromethene 630/650 (BDP 630/650) azide (Lumiprobe) were reacted with DOPE‐DBCO in chloroform to generate DOPE‐630/650. Successful conjugation was validated via thin‐layer chromatography which indicated <1% free dye.

### Generation of Detergent/Lipid Mixture

Lipid stock solutions were made in chloroform and them measured into glass vials and left drying on a desiccator overnight. For solubilization in MEGA‐10 micelles, a 10% MEGA‐10 solution was made in deionized water. The 10% MEGA‐10 solution was then added to the dried lipids and left in a water bath sonicator at 50 to 60 °C until all lipids were solubilized. The lipid/detergent mixture was allowed to equilibrate at room temperature prior to dilution.

### Synthesis of Nanoparticles via Dilution

The lipid/detergent micelles were diluted by rapidly adding buffer to the micelles to reach the target detergent concentration. The samples were then allowed to equilibrate at 25 °C.

### Purification via TFF

Generally, 5 mg of the lipid nanoparticle mixtures were diluted to the target MEGA‐10 concentration and left to self‐assemble overnight. Then samples were diluted to a minimum of 0.02% MEGA‐10 to ensure minimal effect of the detergent on the NPs. Samples were then placed on a KrosFlo KR2i TFF system (Repligen) system using either a 50 kDa molecular weight cutoff (MWCO) mPES membrane with a surface area of 75 cm^2^ (D02‐E050‐10‐N) for particles <100 nm or a 100 kDa MWCO mPES membrane with a surface area of 115 cm^2^ for particles >100 nm (D02‐E100‐05‐N). Samples underwent 10 diafiltration volumes against the buffer used for their self‐assembly. For CND preparations, samples were sterile filtered via a 0.1 µm filter (Cytiva Acrodisc).

### Layer by Layer (LbL) Deposition

LbL‐lisCND particles were generated by mixing lisCND particles at 0.5 mg mL^−1^ lipids with 5 weight equivalents (wt. eq.) of polymers in 10 mm HEPES. After polymer mixing, particles were allowed to incubate on ice for at least 30 min before TFF purification as described previously.^[^ ^43^ ^]^ LbL‐CND particles were generated by first buffer exchanging the sample samples into 50 mm HEPES and 40 mm NaCl via TFF. Then particles at 0.5 mg mL^−1^ were mixed with 5 wt. eq. of PLR in deionized water. After PLR deposition, samples were incubated for 30 min on ice and then purified by TFF into deionized water. After purification, PLR‐coated CNDs at 0.5 mg mL^−1^ lipids were mixed with 5 wt. eq. PLE, incubated for 30 min on ice, and then purified by TFF into deionized water. Control LbL‐liposomes for either lisCND or CND were generated using the same conditions. PLR purification was performed on a 30 kDa mPES membrane with a surface area of 20 cm^2^ (C02‐E030‐05‐N, Repligen) while PLE purification was performed on a 50 kDa mPES membrane with a surface area of 75 cm^2^ (D02‐E050‐10‐N, Repligen). Purification was completed once samples underwent 10 diafiltration volumes. Fluorescently‐labeled lisCNDs contained 0.2 mol% of DOPE‐630/650 while CNDs contained 1 mol% of DSPE‐cy5 (Avanti).

### Characterization of Particle Preparations

Dynamic light scattering (DLS) and zeta potential measurements were made on a Zetasizer Nano ZSP (Malvern). Nanoparticle micrographs were acquired using Transmission Electron Microscopy (TEM) on a JEOL 2100F microscope (200 kV). For cryo‐TEM, particles were buffer exchanged into deionized water via either dialysis or TFF. The microscopes were with a magnification range of 10000‐60,000X. Cryo‐TEM micrographs were analyzed on ImageJ to measure particles diameter. Particles with 1 mol% DSPE‐cy5 were characterized on a Wyatt Dyna Pro Plate Reader.

### In Vitro CND Fluorescently Labeled Lipid Stability

CNDs with 1 mol% of DSPE‐cy5 were mixed with 100% fetal bovine serum (FBS) at 0.1 mg mL^−1^ and left shaking at 37 °C. After 24 h of incubation, any free dye was separated from CNDs on a 100 kDa centrifugal filter (Pall Nanosep) through centrifugation for 15 min at 1000 rcf. Particle fluorescence on the permeate fraction was compared to the total particle fluorescence on a fluorescence plate reader.

### In Vitro Colloidal Stability of CNDs and LbL‐CND

CND and LbL‐CNDs were incubated in 15 mm HEPES (pH 7.2) with 150 mm sodium chloride with 10% FBS or directly mixed with 100% FBS at 10 µg mL^−1^. For LbL‐CNDs, particles were also incubated in 15 mm HEPES (pH 7.2) with 150 mm sodium chloride devoid of FBS. Samples were left mixing in a shaker at 37 °C and DLS size measurements were evaluated at 0, 5, and 24 h.

### Cell Culture

OV2944‐HM‐1 cells (female origin) were acquired through Riken BRC and were cultured in α‐MEM (Gibco) while MC38 (female origin, a gift from the laboratory of Karl Dane Wittrup) were cultured in DMEM (Corning). Cell media was also supplemented with 10% FBS and penicillin/streptomycin with cells incubated in a 5% CO2 humidified atmosphere at 37 °C. All cell lines were murine pathogen tested and confirmed mycoplasma negative by Lonza MycoAlert Mycoplasma Detection Kit. Passage number was maintained below 20.

### In Vitro Cellular Association

The day before dosing, HM‐1 cells were plated on a tissue‐culture 96‐well plate at a density of 50 k cells per well. The next day, wells were dosed with NPs to 0.05 mg mL^−1^ and left for the target incubation time (4 or 24 h). For analysis of association, the supernatant was removed from the well and diluted 10X with DMSO. Cells were then washed three times with PBS then dissolved with DMSO. Fluorescence of NPs associated with cells was then normalized to supernatant fluorescence. The relative fluorescence of each formulation was then compared to an unlayered liposome control containing the same fluorophore. For confocal imaging, 8‐well chambered coverglass (Nunc Lab‐Tek II, Thermo Scientific) were treated with rat tail collagen type I (Sigma–Aldrich) per manufacturer's instructions. HM‐1 cells were plated onto wells at a density of 10k/well and left to adhere overnight prior to NP treatment. After the desired incubation time with NPs, cells were washed 3x with PBS. After washing, cells were fixed in 4% paraformaldehyde for 10 min then washed (3x with PBS) and stained with wheat germ agglutinin (WGA) conjugated to Alexa Fluor488 (Invitrogen) and hoechst 33 342 (Thermo Scientific) following manufacturer instructions. Images were analyzed using ImageJ. Slides were imaged on a Olympus FV1200 Laser Scanning Confocal Microscope.

### CND Cytotoxicity

HEK293 cells were seeded in 96‐well plates at a density of 10 000 cells per well in 100 µL of culture medium (DMEM supplemented with 10% FBS and 1% P/S) and allowed to adhere overnight. The next day, the media was gently aspirated and replaced with 90 µL of fresh media. CND stock solutions (10 µL) were then added to each well and mixed using an Integra pipette. The cells were incubated for 72 h, after which cell viability was assessed using the CellTiter‐Blue assay (Promega) according to the manufacturer's protocol. Viability was normalized to cells treated with 10 µL of deionized water, and MG132 (40 µm) was used as a positive control for cell death.

### Mice

C57Bl/6 and B6C3F1 mice were purchased from Jackson Laboratories. Female mice were used between 8–12 weeks of age unless otherwise noted with weights of 20–25 g. All animal work was conducted under the approval of the Massachusetts Institute of Technology Division of Comparative Medicine (Committee on Animal Care protocol number 2303000488) in accordance with federal, state, and local guidelines. Subcutaneous, intravenous, and intraperitoneal injections were performed with volumes of 100, 100, and 200 µL, respectively. Mice were randomly divided prior to treatments. Before mouse treatments or imaging, mice were anesthetized with 2–3% isoflurane.

### Subcutaneous Tumor Model

C57Bl/6 mice were implanted with subcutaneous MC38 tumors by injecting 10^6^ cells into the right flank. One week after tumor inoculation, mice were injected intravenously via the tail vein with NPs containing 1 mol% DSPE‐cyanine5 (1 nmol dye injected per mouse). In vivo tumor radiant efficiency was measured on an In Vivo Imaging System Spectrum CT (IVIS, Perkin Elmer), and serum was collected via cheek bleeds. After the final timepoint (24 h), mice were euthanized and the major NP clearance organs—liver and spleen—as well as tumor were removed and had their radiant efficiency measured ex vivo by IVIS. Data were analyzed using Living Image software. Background fluorescence measurements were made for each organ based on signal from mice treated with PBS. Recovered fluorescence radiant efficiency was calculated as described previously.^[^ ^40^ ^]^

### Intraperitoneal Ovarian Cancer Model

B6C3F1 mice were inoculated with firefly luciferase‐expressing OV2944‐HM1 (HM‐1‐luc) cells through intraperitoneal (i.p.) injection of 10^6^ cells in PBS. Two weeks after tumor inoculation, mice were injected with 0.75 nmol of 1 mol% DSPE‐cy5 NPs. Analysis was done similar to subcutaneous tumor model with the exception that peritoneal radiant efficiency was measured instead of tumor fluorescence. For correlation analysis, the weight‐normalized bioluminescence flux (p/s/g) and radiant efficiency ([p/s] / [µW/cm^2^]/g) for each organ (excluding main tumor) were analyzed on Graphpad Prism 9 for their correlation via the Pearson's coefficient.

### Statistical Analysis

GraphPad PRISM 10 was used to perform statistical analyses. Comparisons between two groups was performed via unpaired t‐tests. For multiple groups or multiple variable analysis, one‐way, or two‐way ANOVAs were used with Tukey's posthoc correction.

### Ethics Approval

All animal work was conducted under the approval of the Massachusetts Institute of Technology Division of Comparative Medicine in accordance with federal, state, and local guidelines

## Conflict of Interest

I.S.P., P.T.H., and D.J.I. are inventors on a provisional patent filed by the Massachusetts Institute of Technology related to this work.

## Supporting information



Supporting Information

## Data Availability

The data that support the findings of this study are available from the corresponding author upon reasonable request.
