# Supplementary material for: Charge‐Stabilized Nanodiscs as a New Class of Lipid Nanoparticles
Source: Adv Mater. 2024 Nov 14;36(52):2408307. doi: 10.1002/adma.202408307 (PMC11681300; doi:10.1002/adma.202408307)
Supplement: Supplementary file 1 — Supporting Information [file ADMA-36-2408307-s001.docx]

**Supplemental Information**

Charge-Stabilized Nanodiscs as a New Class of Lipid Nanoparticles

*Ivan S. Pires, Alexander Hostetler, Gil Covarrubias, Isabella S. Carlo, Jack R. Suggs, B.J. Kim, Andrew J. Pickering, Ezra Gordon, Darrell J. Irvine*, Paula T. Hammond**


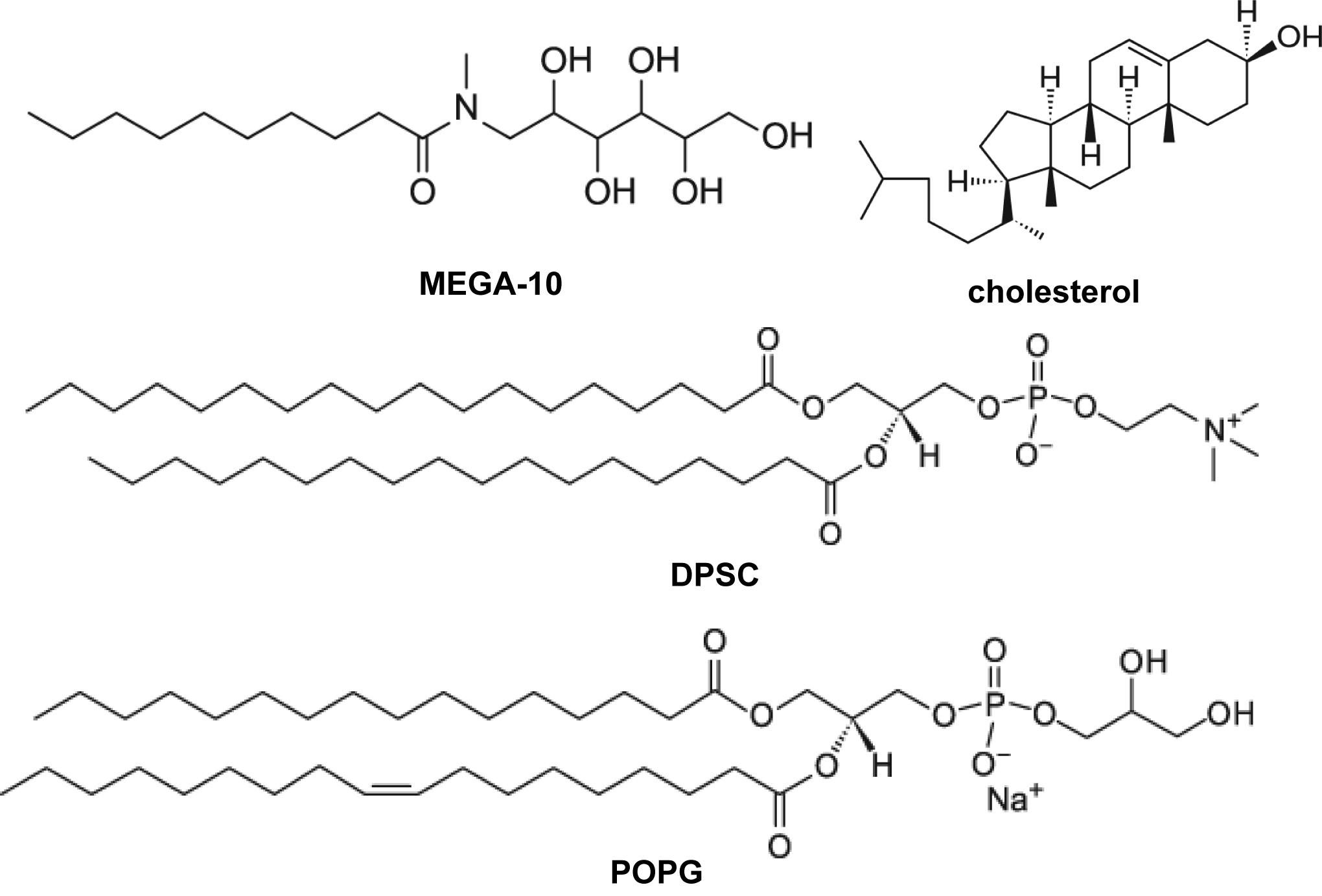
**Figure S1**. **Chemical structure of components used for particle self-assembly.** Shown are structures for n-Decanoyl-N-methyl-D-glucamine (MEGA-10), cholesterol, 1,2-distearoyl-sn-glycero-3-phosphocholine (DSPC), and 1-palmitoyl-2-oleoyl-sn-glycero-3-phospho-(1'-rac-glycerol) (sodium salt) (POPG).


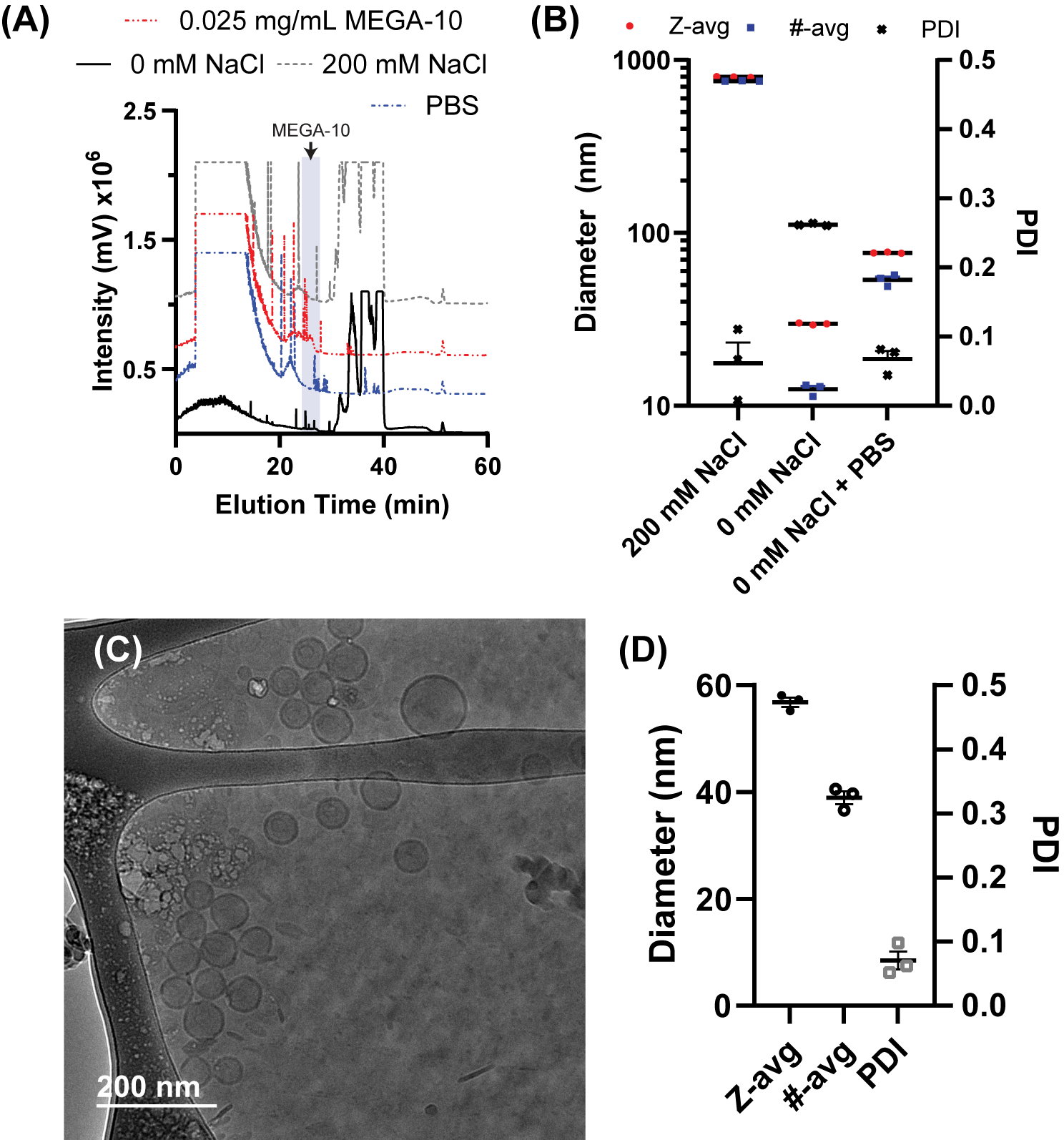


**Figure S2**. **Characterization of lisCNDs and high POPG composition diluted with PBS.** **(A)** Representative chromatograms from high-pressure liquid chromatography (HPLC) coupled with an evaporative light scattering detector (ELSD) for PBS only, 0.025 mg/mL sample in PBS, and samples allowed to be assembled at 0.1% MEGA-10 in 0 mM NaCl or 200 mM NaCl buffers then purified via TFF. **(B)** Z-avg, #-avg and PDI of purified samples allowed to assemble at 0.1% MEGA-10 in 200 mM NaCl, 0 mM NaCl, or 0 mM NaCl then exposed to 1X PBS after purification. **(C)** CryoTEM micrographs of purified samples from dilution of lipid/detergent micelles using 0 mM NaCl, but then added 1X PBS. **(D)** Size and PDI of lipid/detergent micelles with 3:3:4 molar ratios of DSPC:cholesterol:POPG diluted to 0.05% MEGA-10 then purified via TFF. Error bars represent SEM.


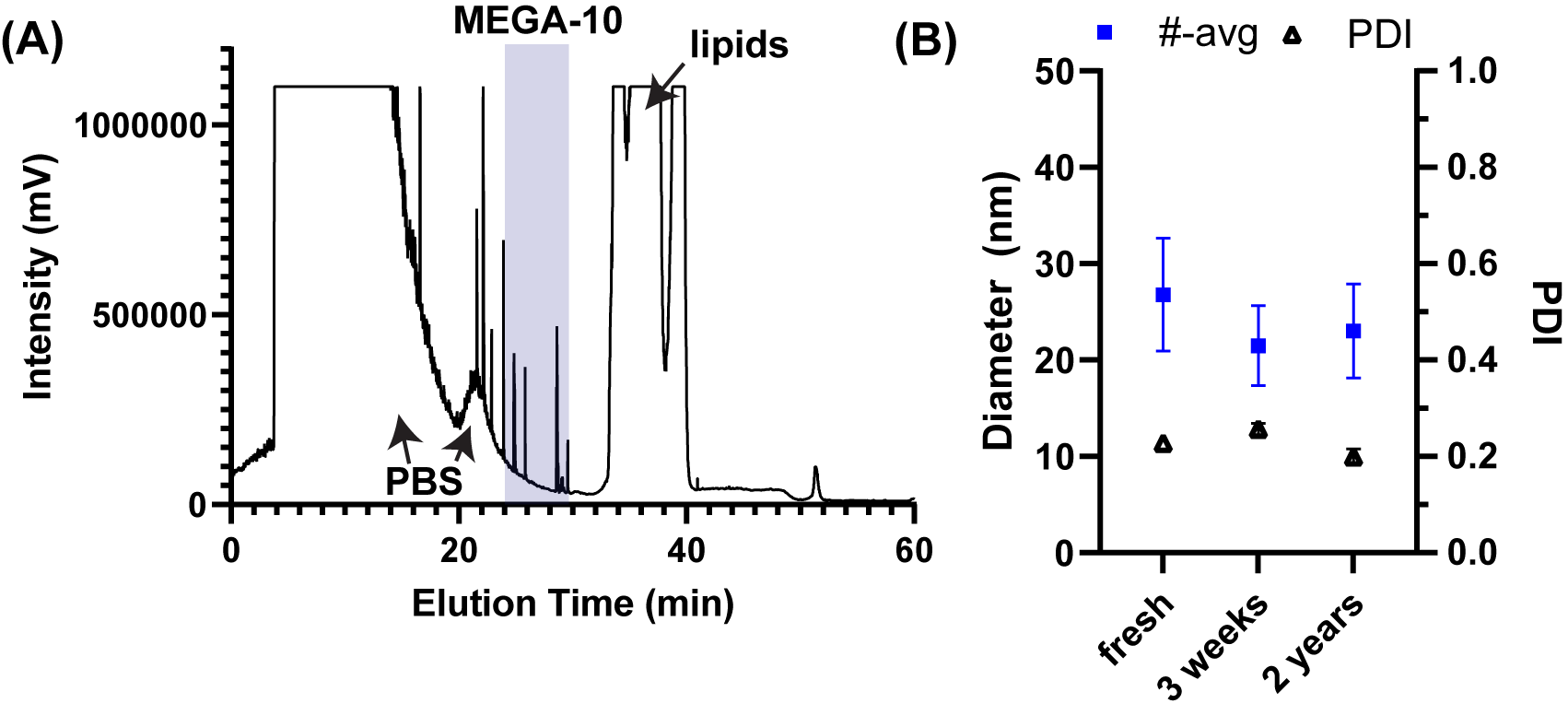


**Figure S3. Purity and long-term stability of CNDs in PBS. (A)** Representative chromatograms from high-pressure liquid chromatography (HPLC) coupled with an evaporative light scattering detector (ELSD) of CNDs post TFF purification. **(B)** DLS Z-avg, #-avg, and PDI of CNDs after purification and after storage in PBS for 3 weeks and 2 years at 4 °C.


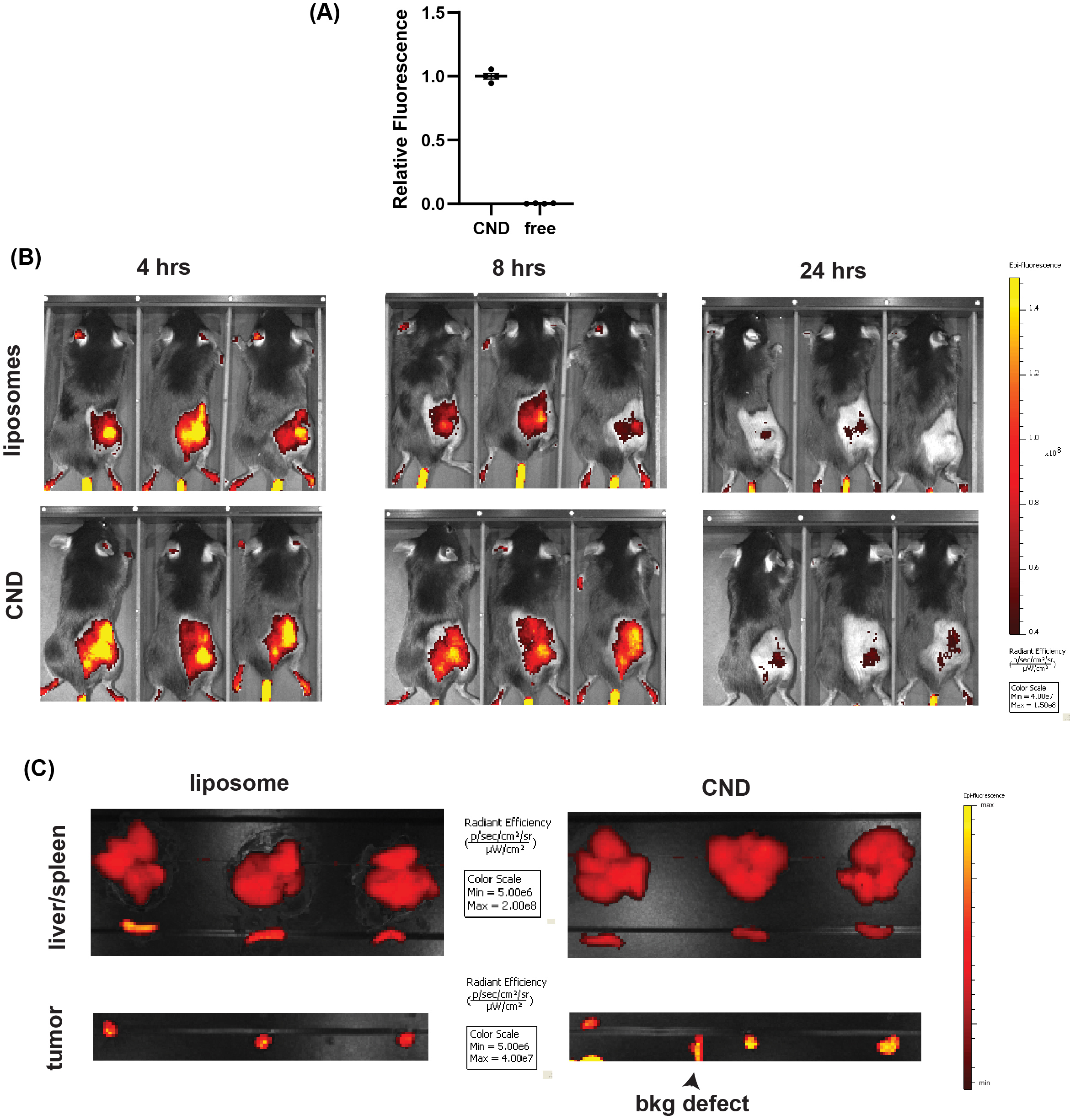


**Figure S4. CNDs show improved subcutaneous tumor accumulation compared to liposomes. (A)** Quantification of free dye released from CNDs with 1 mol% DSPE-cy5 incubated in 100% FBS for 24 hrs at 37 °C. **(B)** IVIS fluorescence images of mice injected intravenously with fluorescently labeled liposomes or CNDs **(C)** Ex vivo IVIS fluorescence images of clearance organs (liver and spleen) and tumors of mice injected intravenously with fluorescently labeled CNDs or liposomes.


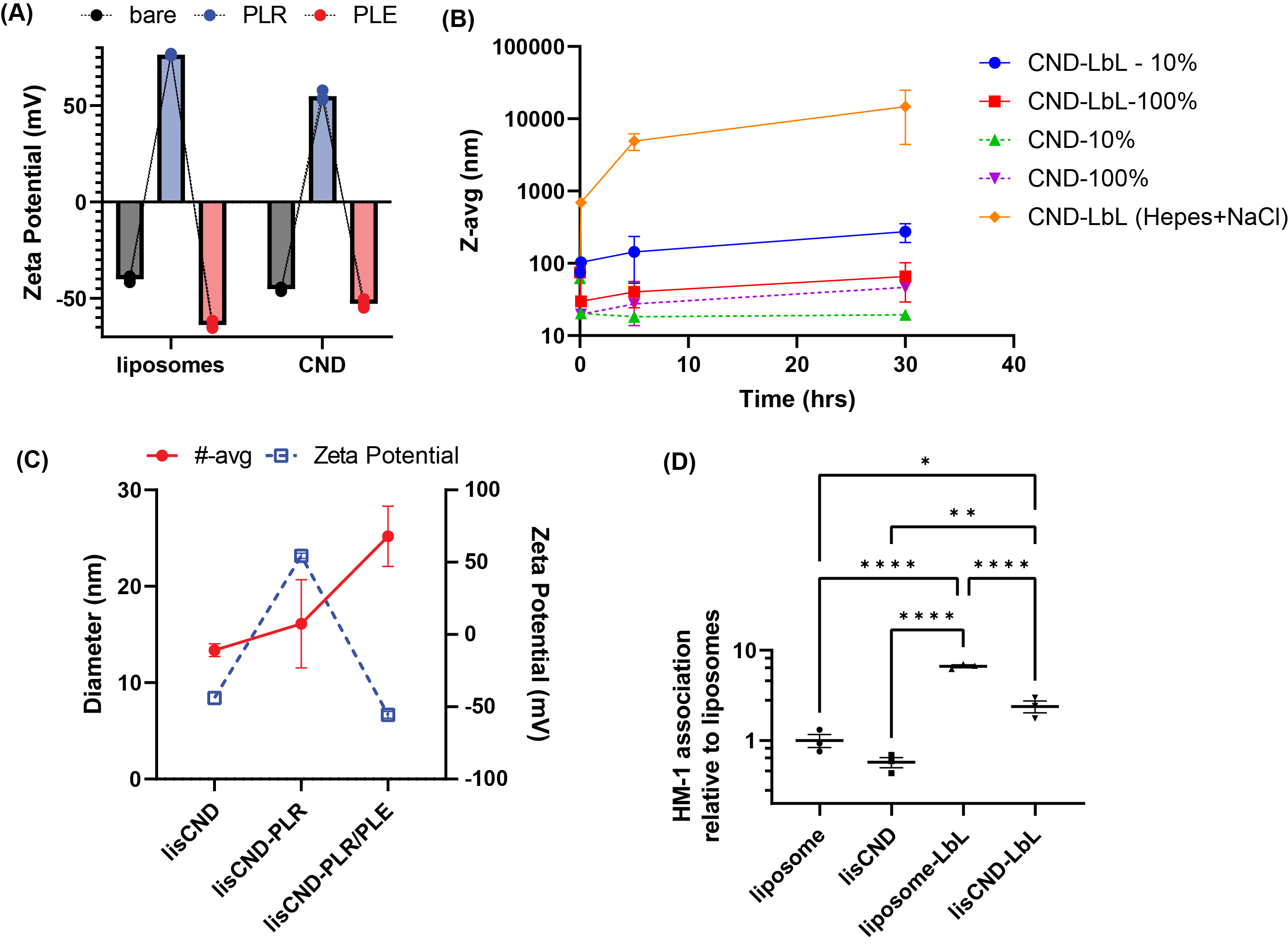


**Figure S5**. **Characteristics of LbL-CNDs and LbL-lisCNDs. (A)** Zeta potential of liposomes and CNDs upon layering with PLR and PLE. **(B)** DLS Z-avg size of NPs incubated in 15 mM HEPES (pH 7.2) and 150 mM NaCl with or without 10% FBS or in 100% FBS. **(C)** Size and zeta potential of lisCNDs upon layering with PLR and PLE. **(D)** In vitro HM-1 association of NPs relative to liposomes 24 hrs after dosing cells. Error bars represent SEM. Statistical comparisons in C was performed using one-way analysis of variance (ANOVA) with Tukey’s multiple-comparisons test. Asterisks denote p-values: ****p < 0.0001, ***p < 0.001, **p < 0.01, *p < 0.05.


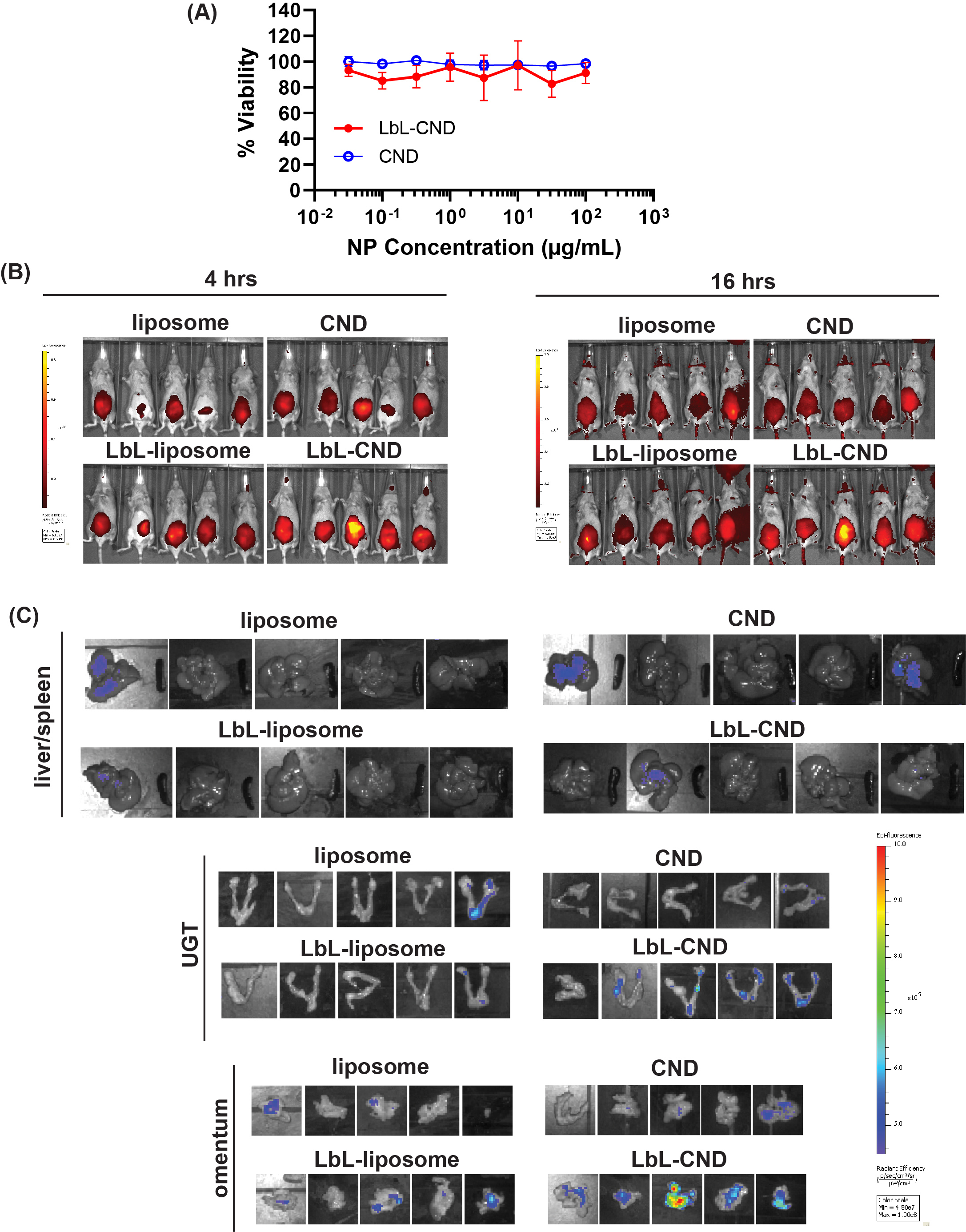


**Figure S6. Cytotoxicity and biodistribution of LbL-CNDs. (A)** Viability of HEK293 cells dosed with increasing concentrations of CND or LbL-CNDs. **(B)** In vivo IVIS images of peritoneal fluorescence from mice bearing HM-1 i.p. tumors dosed with liposomes, LbL-liposomes, CNDs, or LbL-CNDs. **(C)** Ex vivo IVIS fluorescence images of clearance organs (liver and spleen) or organs with high tumor burden (omentum and UGT) from mice bearing HM-1 tumors dosed with either liposomes, LbL-liposomes, CNDs, or LbL-CNDs.
